# P-1487. β-tricalcium Phosphate/Calcium Sulfate Loaded with Contezolid Acefosamil (MRX-4) for Antimicrobial Potency, Prevention and Killing Efficacy of MRSA Biofilm

**DOI:** 10.1093/ofid/ofae631.1657

**Published:** 2025-01-29

**Authors:** Nan Jiang, Peng Chen, Haoyang Wan, Guanqiao Liu, Qinrong Lin, Bin Yu

**Affiliations:** Nanfang Hospital, Southern Medical University, Guangzhou, Guangdong, China (People's Republic); Nanfang Hospital, Southern Medical University, Guangzhou, Guangdong, China (People's Republic); Nanfang Hospital, Southern Medical University, Guangzhou, Guangdong, China (People's Republic); Nanfang Hospital, Southern Medical University, Guangzhou, Guangdong, China (People's Republic); Nanfang Hospital, Southern Medical University, Guangzhou, Guangdong, China (People's Republic); Nanfang Hospital, Southern Medical University, Guangzhou, Guangdong, China (People's Republic)

## Abstract

**Background:**

The use of local antibiotics loaded by antibiotic carriers represents an important strategy for the treatment of osteoarticular infections. This study evaluated the antimicrobial duration of potency and the prevention and killing efficacies of contezolid acefosamil (MRX-4) against MRSA biofilm in *in-vitro* assays.

**Figure 1:**
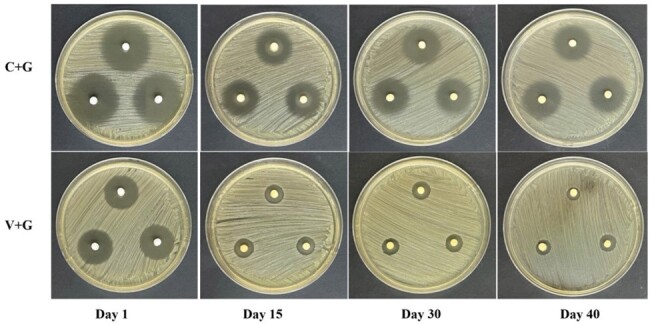
ZOIs of C+G versus V+G loaded by CS

**Methods:**

The elution kinetics of contezolid acefosamil (C) and vancomycin (V) in combination with gentamicin (G), loaded with either β-tricalcium phosphate/calcium (β-TCP/CS) sulfate or CS, in the presence of an MRSA (ATCC43310) infection was assessed. The efficacies of the agents in preventing biofilm formation and killing pre-formed biofilms were assessed using colony-forming unit count and confocal laser scanning microscopy.

**Figure 2:**
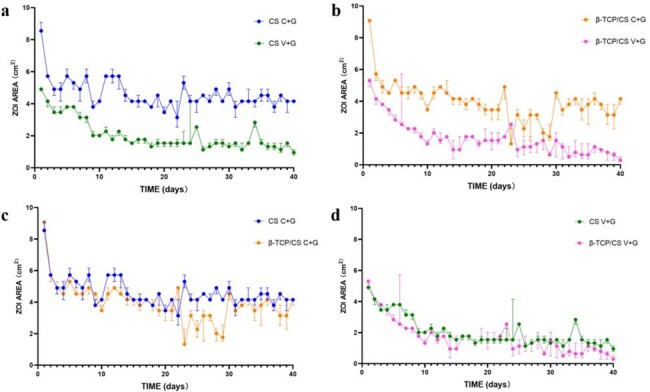
Changes of ZOIs of C+G versus V+G (a, b) and β-TCP/CS versus CS (c, d)

**Results:**

In a repeated Kirby-Bauer like zone of inhibition (ZOI) assay, V+G showed potency against MRSA for approximately 40 days. While the ZOIs of C+G at 40 days were similar to those at 15 days, which were significantly larger than those of V+G (*P* < 0.05). In the MRSA biofilm prevention assays, both C+G and V+G showed similar efficacies in preventing bacterial colony formation, with approximately 8 logs lower colony-forming units than those in the control group at 1 and 3 days (*P* < 0.01). The killing efficacies on pre-formed biofilms showed significant decrease of approximately 3 to 4 logs at 1 and 3 days following treatment with C+G and V+G (*P* < 0.001). Furthermore, the confocal laser scanning microscopy results supported the colony-forming unit outcomes. Additionally, β-TCP/CS and CS revealed similar antimicrobial potency and prevention and killing efficacies in these *in-vitro* assays.

**Figure 3:**
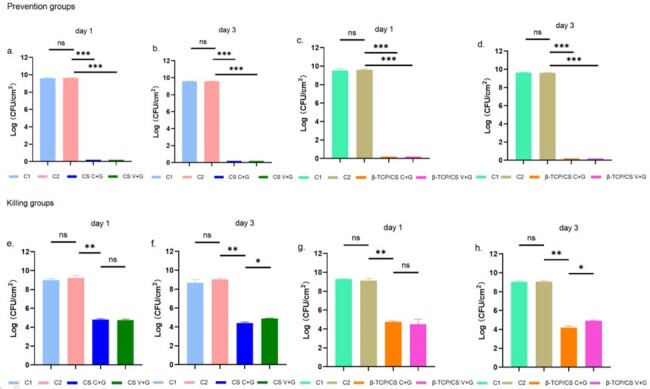
Colony-forming units in the prevention and killing groups (C1:control group 1: blank groups, C2: control group 2: β-TCP/CS or CS beads without antibiotics; ns: not statistically significant, *P ＜ 0.05, **P ＜ 0.01, ***P ＜ 0.001 )

**Conclusion:**

The present study demonstrated that contezolid acefosamil displays similar prevention and killing efficacies against MRSA biofilms in comparison to vancomycin. However, the antimicrobial duration of potency of contezolid acefosamil is longer than vancomycin. Further clinical trials are required to determine whether contezolid acefosamil can be locally applied for the treatment of osteoarticular infections.

**Disclosures:**

**All Authors**: No reported disclosures

